# Supplementary material for: Wnt/β-Catenin Signaling Contributes to Paclitaxel Resistance in Bladder Cancer Cells with Cancer Stem Cell-Like Properties
Source: Int J Mol Sci. 2021 Dec 31;23(1):450. doi: 10.3390/ijms23010450 (PMC8745426; doi:10.3390/ijms23010450)
Supplement: Supplementary file 1 [file ijms-23-00450-s001.zip › Figure S1.pdf]

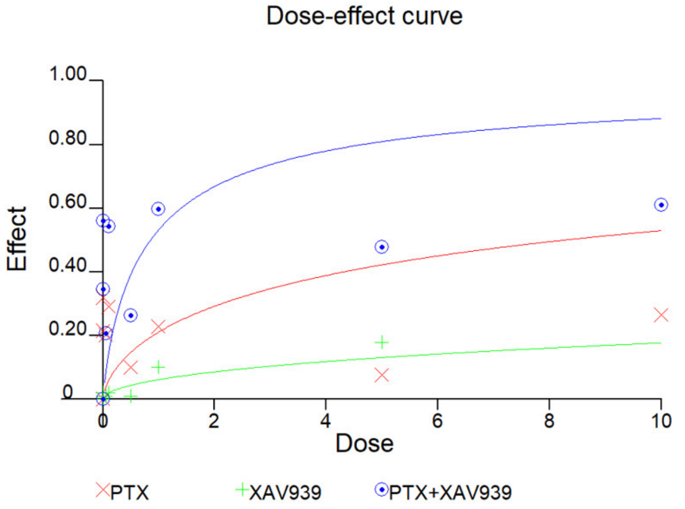

Figure S1. Calculation of combination index (CI) of combined treatment of paclitaxel (PTX) and XAV939 in bladder cancer cell line HT1197 using Calcusyn. Dose-effect curve for HT1197 cells, with a CI of 0.10685.
