# Supplementary figures and images for: Wnt/β-Catenin Signaling Contributes to Paclitaxel Resistance in Bladder Cancer Cells with Cancer Stem Cell-Like Properties
Source: Int J Mol Sci. 2021 Dec 31;23(1):450. doi: 10.3390/ijms23010450 (PMC8745426; doi:10.3390/ijms23010450)

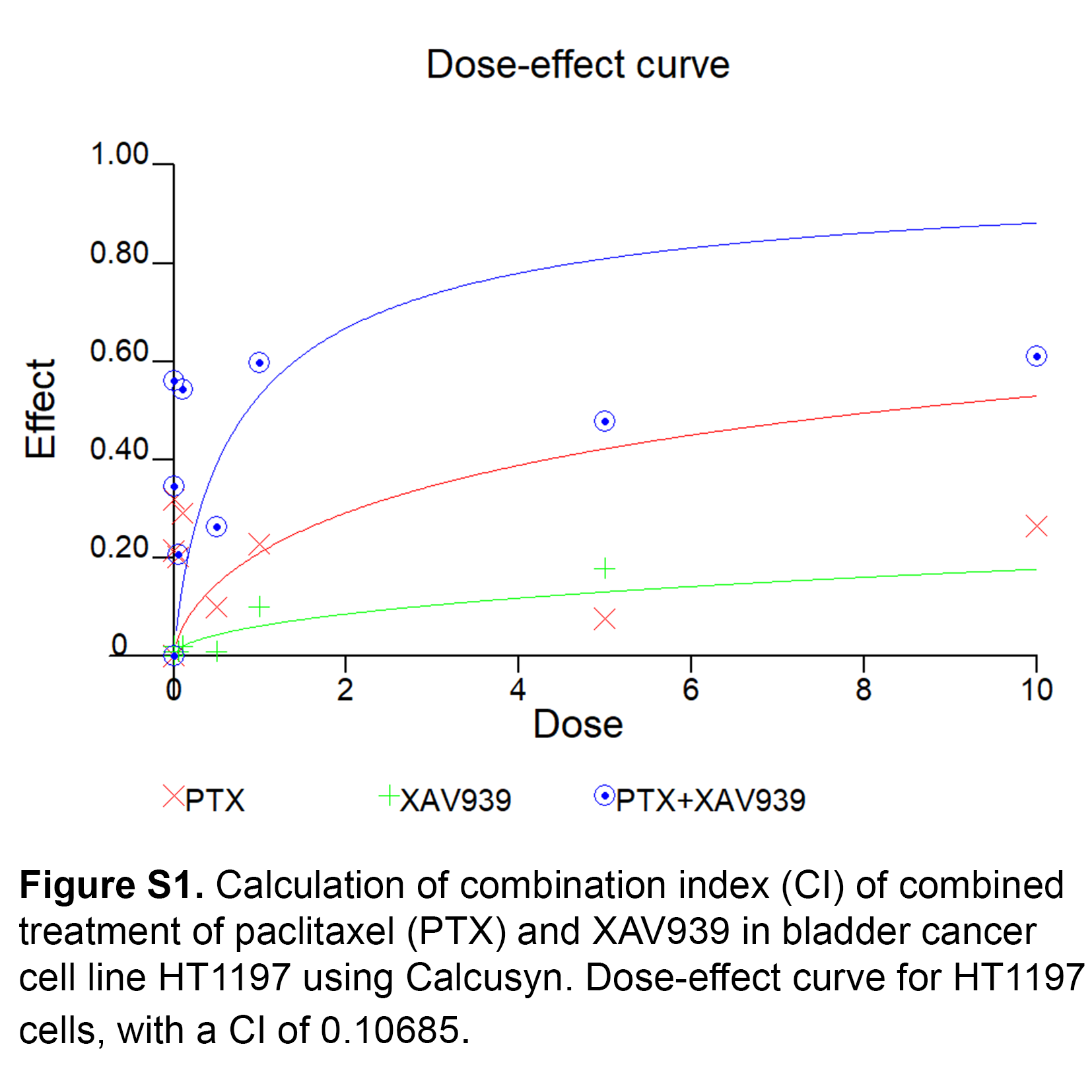

Supplement: Supplementary file 1 [file ijms-23-00450-s001.zip › Figure S1 with caption.tif]
